# No. 3. Cases and Dissections

**Published:** 1808-12

**Authors:** Thomas Beddoes


### CASES AND DISSECTIONS,

By Thomas Beddoes, M. D.

A Friend of mine had three noble opportunities of verifying the sound and natural state of the brain; and as he has seen many dissections, his report ought to have much weight.

C. D. received a wound from a very small file sharpened to a point. His respiration, when my friend saw him, was difficult and hurried. His eyes staring, and his countenance anxious. Bled with relief to the dyspnœa, which soon returned with restlessness and anxiety; pain in back; numbness in right hand and arm; a second bleeding relieved for a few minutes; perfectly sensible till cold sweats came on; and the scene closed in four hours from the accident. The instrument had entered the right side of the thorax, about an inch and a half below the nipple, slanting towards the sternum, and from the intercostal muscles between fifth and sixth ribs, passed through the right lobe of the lungs, entered the pericardium, and wounded the ventricle, dividing the coronary vein and artery. The pericardium was filled with coagulated blood.

Dura mater not more than naturally attached; vessels smaller than I have usually found them, and the pia mater less vascular. Cerebrum not hard, but it had an elastic firmness; cut beautifully white, not having any appearance of those red points usually found. The ventricles empty, and almost dry.

In

In two others, one of whom died on the spot, the other the 6th day, from wounds of the chest, the appearances of the brain were exactly similar.

Such cases, I should think, give truer means of judging of the real state of the inflamed and uninflamed brain, than all the arts of anatomy.

It seems to me, that facts which I have formerly quoted, others related in the former papers, and some which I have seen since, establish a curious pathological consent between the brain and stomach. In various instances, an inflammation in the upper part of the stomach shall arise, when the brain with its appendages are turgid with blood in an extreme degree, or inflamed; and the stomach shall sometimes exhibit no pain or other sign of inflammation. This sympathy seems alike, whether the brain suffer from external or internal causes. Dr. Haen, as I have observed in my *Researches on Fever*, touched with a red hot iron the scull of a boy affected with amaurosis, and with periodical vomiting. The boy eat a good supper on the fourth day; had periodical vomiting, and died in six hours after his supper, with intense inflammation of his brain, and the cardia even gangrenous in two spots; prodigious adhesion of the lungs; as also was the case in a girl likewise cauterized, but whose stomach shewed no inflammation.

4th Case in your last Number, the head and stomach highly inflamed, though not exclusively.

5th Case, ditto ditto, intense inflammation of both head and stomach, though not exclusively confined to those parts.

Case 6. The brain must be considered as too full of blood here, though without intense marks of inflammation; how violent the inflammation of the cardia?

Case 8 speaks strongly for itself, where, in all that intensity of inflammation of the stomach, there was no sickness in the morning, or while the patient was sensible.

A patient, æt. 19, whose case I shall quote particularly on another occasion, lingered for several weeks under symptoms, without any apparent reference to the head, asked very early one morning for some water. The water was drank very comfortably; I ascertained this fact accurately from the attendants. The patient lay down. In about half an hour, alarming state of respiration, and almost sudden death.

The examination of the body was made in the course of the day. The head was prodigiously turgid with blood, and the cardia besides exclusively inflamed. There were stripes along along the stomach, leading up to the cardia, and when you pressed ever so gently upon the red spots, visible in the stripes, blood oozed. Here we have full assurance of the stomach at ease half an hour before death, and yet so inflamed on recent examination of the stomach.

A girl, æt. 13, was lately visited by my friend Dr. C. In a few hours I joined him. He, from preceding torpor and other symptoms, suddenly succeeded by insensibility, judged the head to be loaded with blood, or that it was a case of Hydrocephalus Internus. I could only assent to the opinion. The arterial system was singularly palsied, and out of all proportion to the loco-motive. The heart laboured excessively.—A number of punctures at the two visits were made, scarce a streamlet of blood flowed from divided vein or branch of the temporal; and when a little blood did flow, it rendered the pulse almost insensible; while the power of swallowing and performing some muscular movements was considerable. Clear as to the origin of the evil, we persevered in our efforts to draw blood; and I doubt not but a good stream of blood would have relieved the arterial system and head, but we persevered without success. We left our patient, expecting her very speedy death. She lingered, however, for thirty-six hours.

An early examination of the body, shewed that state of the head which we expected. It was every where gorged with blood, and near two ounces of lymph were found in the lateral ventricles, besides more within the membranes.

I had said during the dissection of the head, seeing such immense accumulation of blood or inflammation in the brain, *that we should find the stomach, especially the upper orifice, inflamed.* Yet this patient had drank or swallowed quietly, even after her insensibility, all that was put into her mouth. All the medical attendants during life were present, with the addition of Mr. King, surgeon, Hot-wells, and his pupil Mr. Delaroche. This is the note of the state of the stomach taken down on the spot. "Stomach inflamed in a blotch near the cardia: some effusion of blood under the villous coat, and dark red spots within; the blotch gave out blood on slight pressure with the finger." The omentum was injected red.

The lungs were full of tubercles, though no alarming cough had ever been noticed.

I mean not to present this (secondary?) inflammation of the stomach as a general law. But I think, whenever the upper part of the stomach is found unexpectedly inflamed, the the head, (a circumstance so often neglected) should be opened too. Perhaps, the real disease of one of our most upright and illustrious diplomatic characters, may be illustrated by the preceding dissections. His able biographer has given the public a glimpse of the unsatisfactory occurrences attending his latter end. At the time of his death, an old and intimate friend related all the particulars to me, and shewed me the written account of the dissection.

Mr. ———— had had frequent attacks of gout during the last six years. The death of Mr. Pitt, "the affairs of Europe damped his spirits.* He had now been confined with flying gout for a couple of months. In this languishing reduced state, he was declared by several of the faculty to be convalescent till three days before his death. His own feelings led him to prefer creams, jellies, and other articles of a cool and softening nature. He was ordered stimulants, and even brandy."

Dissection shewed the gullet terminating in the stomach extremely vascular, and the internal membrane itself in an abraded state. This appearance extended about an inch into the œsophagus, and two inches along the coats of the stomach. Internal membrane of the right kidney unusually vascular.

In this Case, may there not seem suspicion that the head was charged with blood, or inflamed?

* Expression in private letter.

